# Proof of biology for amyloid plaque lowering therapies in pre‐clinical models of Alzheimer’s Disease

**DOI:** 10.1002/alz70859_100185

**Published:** 2025-12-25

**Authors:** Choya Yoon

**Affiliations:** ^1^ Merck & Co., Boston, MA USA

## Abstract

**Background:**

Trem2 gain‐of‐function therapeutic approaches are actively being pursued as immune‐stimulatory, disease‐modifying strategies in the clinic.

**Method:**

We developed robust pharmacological tools to selectively stimulate and dissect pathway signaling and determined the extracellular activation profile with the goal of identifying genetically‐implicated cytokine signatures in multiple cell systems. Through a rigorous genetic and pharmacological validation strategy, we identified a conserved fingerprint of chemokine secretion. Translatability of chemokine biomarkers in preclinical models of neurodegeneration was interrogated by profiling cytokine signatures in brain tissue from mice with amyloid pathology, tauopathy, or chemically‐induced CNS demyelination using an innovative multiplexing immunoassay that enables simultaneous measurement of up to 70 targets in a single analysis.

**Result:**

We showed that a robust upregulation of a Trem2‐dependent chemokine biomarker signature was consistent across different mouse models establishing strong in vitro and in vivo agreement. For preclinical pharmacological validation of the defined chemokine signature as PKPD endpoints, a highly potent Trem2 mouse surrogate Trem2 antibody was acutely dosed; a robust, dose‐dependent brain chemokine response in Trem2‐expressing WT mice as well as plaque‐burden mice was found, while this response was abolished in Trem2‐deficient mice, thus providing further evidence for target‐specific induction of selected chemokine biomarkers triggered by Trem2 signaling in activated microglia.

**Conclusion:**

The current data therefore describe novel biomarker and functional insights into a key microglial pathway implicated in human disease and furthermore lay the framework for a translational biomarker strategy to support progression of Trem2 therapies for clinical development.

